# Ni-modified magnetic nanoparticles for affinity purification of His-tagged proteins from the complex matrix of the silkworm fat body

**DOI:** 10.1186/s12951-020-00715-1

**Published:** 2020-11-06

**Authors:** Robert Minkner, Jian Xu, Kenshin Takemura, Jirayu Boonyakida, Hermann Wätzig, Enoch Y. Park

**Affiliations:** 1 Department of Bioscience, Graduate School of Science and Technology, Shizuoka University, 836 Ohya, Suruga-ku, Shizuoka, 422-8529 Japan; 2 Institute of Medicinal and Pharmaceutical Chemistry, TU Braunschweig, Beethovenstr. 55, 38106 Braunschweig, Germany; 3 Laboratory of Biotechnology, Green Chemistry Research Division, Research Institute of Green Science and Technology, Shizuoka University, 836 Ohya, Shizuoka, 422-8529 Japan; 4 Institute of Biology and Information Science, Biomedical Synthetic Biology Research Center, School of Life Sciences, East China Normal University, Shanghai, 200062 People’s Republic of China

**Keywords:** Magnetic nanoparticles, Affinity purification, Recombinant protein, Silkworm, Magnetic separation

## Abstract

Purification of recombinant proteins is often a challenging matter because high purity and high recovery are desired. If the expressed recombinant protein is also in a complex matrix, such as from the silkworm expression system, purification becomes more challenging. Even if purification from the silkworm expression system is troublesome, it benefits from a high capacity for the production of recombinant proteins. In this study, magnetic nanoparticles (MNPs) were investigated as a suitable tool for the purification of proteins from the complex matrix of the silkworm fat body. The MNPs were modified with nickel so that they have an affinity for His-tagged proteins, as the MNP purification protocol itself does not need special equipment except for a magnet. Among the three different kinds of investigated MNPs, MNPs with sizes of 100 nm to 200 nm and approximately 20 nm-thick nickel shells were the most suitable for our purpose. With them, the total protein amount was reduced by up to at least approximately 77.7%, with a protein recovery of around 50.8% from the silkworm fat body. The minimum binding capacity was estimated to be 83.3 µg protein/mg MNP. Therefore, these MNPs are a promising tool as a purification pretreatment of complex sample matrices.
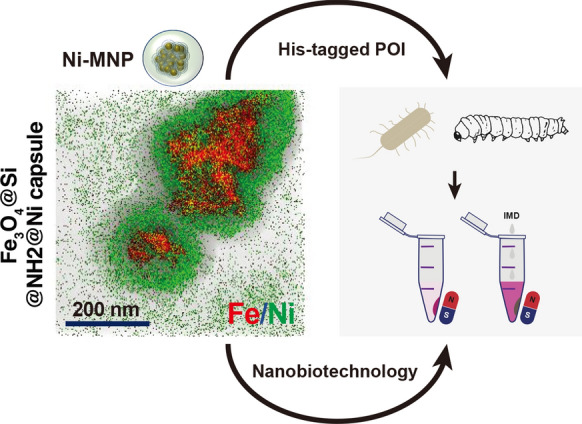

## Introduction

Usually, for protein production, *Escherichia coli* or yeast systems are mainly utilized because they are already well established, and their advantages are known. However, low protein quality or the inability to generate posttranslational modifications are typical disadvantages [1]. However, another interesting option for us is using the larvae of the domestic silkworm, *Bombyx mori,* as a recombinant protein expression system. Because this system is a eukaryotic insect cell system, the silkworm expression system provides the same posttranslational modifications as insect cell lines. For specific proteins, an even higher production yield than achieved with the systems mentioned above can be achieved [1–6]. In the system, the injected recombinant *Bombyx mori* nucleopolyhedrovirus (BmNPV) bacmid infects the larvae's cells, which then start to express the desired recombinant proteins. Depending on the expressed protein, the protein will remain inside the cells or released into the hemolymph [2].

The silkworm contains abundant host proteins. These proteins make purification from the silkworm extremely challenging [1]. Moreover, lipid content in silkworm tissues, such as the fat body, is also abundant compared to that in other hosts used for protein expression [7, 8]. Usually, for purification from this system, traditional sucrose gradient density centrifugation and affinity purification are used, but generally, they have not been explored for optimization [9–11]. Approaches to improving purification with this system solely based on standard methods also present challenges [12]. Currently, no broadly practical purification approach is available for the purification of proteins from silkworms, except for some exceptional cases in which the host proteins are abundant and diverse. The silkworm expression system is an example of a complex sample matrix. Therefore, purification from this system is still a bottleneck for the use of this expression system as it was already reviewed [7], and we could show that the purification is indeed not easy [12, 13]. Therefore, we will not discuss this aspect exhaustively any further in this paper.

Nanoparticles or magnetic nanoparticles (MNPs) are currently an emerging technology in many scientific fields. These nanomaterials display several advantages because of their nanosize, which is approximately 10–100 nm. They can be used for magnetic resonance imaging, targeted drug delivery, targeted destruction of cancer through hyperthermia, magnetic transfection, tissue engineering, or purification [14, 15]. MNPs can be produced through different methods, including grinding, thermal decomposition, microemulsion, chemical vapor deposition, coprecipitation, a reaction in a constrained environment, the polyol method, flow-injection synthesis, and sonolysis [14, 16–18]. Methods for further surface modification have not been incorporated yet and are performed to modulate solubility, stability, internalization, or toxicity [14, 16, 17, 19]. One of the most commonly used coating materials utilizes silica because of its efficiency, reduced toxicity, aggregation prevention, and hydrophilicity [14, 19]. In terms of toxicity, it is commonly assumed that iron nanoparticles are noncytotoxic and safe for use at a concentration of 100 µg/ml [16, 20]. Moreover, they can be cleared by the endogenous iron metabolic pathway, leading to incorporation into hemoglobin [16]. The relative non-toxicity makes iron nanoparticles attractive not only for medical purposes but also for downstream biotechnological processes because they don't need to be removed under certain circumstances in some products.

It is then reasonable to assume that MNPs are a viable option to purify recombinant proteins. Purification can be performed in two ways: direct binding of the target protein or by removing unwanted proteins. It was shown that the latter could be a safe and effective way to remove haze-forming proteins from wines [21]; the surfaces of the MNPs were modified with an amine, carboxyl, or oxazoline functional groups. Silica-coated iron magnetic nanoparticles functionalized with a nickel shell were used to remove the abundant protein hemoglobin from bovine blood [19]. Nickel has a high affinity for the His-tag and proteins with a high number of histidine residues. Therefore, bovine hemoglobin was successfully and selectively reduced, even if the level of reduction in the diluted bovine blood sample was low due to protein–protein interactions [19].

Another method involves modification of the MNPs to such an extent that they can specifically separate the target protein. These modifications do not necessarily need to be antibodies, as adamantane beta-cyclodextrin (β-CD) was used to modify MNPs, which were then able to bind the target lectin [22]. This construct was able to separate concanavalin A from a mixture containing peanut agglutinin. The elution was performed by adding mannose as a competitive ligand [22]. Ta et al. [23] modified MNPs in a modular fashion so that they were able to separate the targeted biomarkers, even if they were displayed on whole eukaryotic cells. This modularity also allows the rapid switching of the receptor on the MNPs to target different biomarkers [23].

Another method involves the design of the protein to enhance compatibility with the MNP. A peptide tag of six glutamates proved to be very useful for binding bare iron oxide nanoparticles (BIONs) [24]. It could even be used for purification on an industrial scale to generate BIONs that could achieve a mean recovery of 81% [24]. Of course, the MNPs can be modified to be compatible with already existing protein tags. Jose et al. [25] encapsulated Fe_3_O_4_ nanoparticles in polystyrene nanoparticles and functionalized them with Ni^2+^-nitrilotriacetic (NTA) to purify a His-tagged model protein. These particles were even more efficient than the already commercially available Ni^2+^-NTA-magnetic beads [25]. In a study similar to our conducted research, bare iron oxide nanoparticles were used, and His-tagged green fluorescent protein (GFP) was purified as a model protein; the most complex sample matrix was an *E. coli* cell lysate [26]. However, the study demonstrated the usage of high-gradient magnetic fishing with an electromagnet, 1 L crude cell lysate, and a system with a capacity of 2 L.

Because such MNPs are such intriguing materials in terms of their uses, they are already commercialized. However, these commercialized products are not cost-effective, especially when purification must be performed on at least a milliliter scale. Therefore, they are constantly further investigated and improved [27–29]. We tried to purify recombinant proteins from complicated and hard-to-purify matrices using a simple and easy pretreatment step to purify His-tagged recombinant proteins. One option to achieve this could be to generate such MNPs in the laboratory when needed. Therefore, our study focused on the preparation of magnetic nanoparticles, which are inexpensive, easily made in the laboratory, and disposable. Three different kinds of particles were tested to determine their usability for purification, and one of them proceeded to be utilized for further investigations. This particle was able to purify a model fluorescent protein from an *E. coli* cell lysate with high purity and to achieve high recovery of the target proteins from the complex silkworm fat body sample as a pretreatment step. Moreover, these nanoparticles have a significantly higher binding capacity than a commercial product with a similar elution capability, as we will show in this study.

## Materials and methods

If not mentioned otherwise, all materials and reagents, such as tetraethyl orthosilicate, FeCl_2_, FeCl_3_, (3-aminopropyl)trimethoxysilane, and Ni(OAc)_2,_ were purchased from Sigma-Aldrich (Tokyo, Japan). Ammonium hydroxide was purchased from Wako Pure Chemical Ind. Ltd. (Osaka, Japan).

### Plasmid preparation and expression of SpyCatcher-mCherry-SpyTag in E. coli

The mCherry DNA fragment was subcloned from the plasmid pmCherry (Catalog # 632522, TakaraBio, Japan) using specific primers (mCherryFw: 5′-gtgagcaagggcgaggaggat-3′; mCherryRv: 5′-cttgtacagctcgtccatgcc-3′) into pFastBac-SpyCatcher/SpyTag [30] and was designated pFB-SC-mCherry-ST. The recombinant plasmid consisted of a His tag, StrepTag II, SpyCatcher, SpyTag, and tobacco etch virus (TEV) protease cleavage sites. The plasmid was used as a template for amplifying the whole gene sequence using primers (Cat-FW: 5′-atgcaccaccaccaccatcaccatcac-3′, pFastBac-RV: 5′-acaaatgtggtatggctgatt-3′) and subsequently subcloned into pET-41a ( +) (Novagen, Tokyo, Japan), and the resulting construct was designated pET41-SC-mCherry-ST. The recombinant plasmid was electroporated into Rosetta-gami 2(DE3) *E. coli* (Novagen, Tokyo, Japan) to express the recombinant mCherry protein.

For the expression of SC-mCherry-ST, *E. coli* Rosetta-gami™ 2(DE3)/pET41-SC-mCherry-ST were inoculated into 1 L of Luria–Bertani medium (LB broth, Invitrogen, Thermo Fisher Scientific, Tokyo, Japan) containing 25 µg/mL kanamycin and incubated at 37 °C until the OD_600_ reached 0.5. Then, protein expression was induced by adding isopropyl β-D-1-thiogalactopyranoside (IPTG) at a final concentration of 0.5 mM, followed by incubation at 16 °C for 16 h. Subsequently, the cells were collected by centrifugation (6000×*g*, 4 °C, 15 min), washed twice with ice-cold phosphate-buffered saline (PBS, pH 7.3), and stored at − 80 °C before use.

### Recombinant B. mori nucleopolyhedrovirus (BmNPV) bacmid preparation for SpCaVP1 and EDIII

The Norovirus VP1 DNA fragment was subcloned into the pFastBac-SpyCatcher plasmid [24] from a recombinant *Autographa californica* nucleopolyhedrovirus (AcMNPV) kindly provided by Dr. Tian-Cheng Li (Department of Virology 2, National Institute of Infectious Diseases, Musashimurayama, Japan). The DNA sequence also encoded poly-tags (His-Strep-TEV-NoroVP1-SpyCatcher) and consisted of a His-tag, a Strep-tag II, a TEV protease cleavage site, and a SpyCatcher. The resulting plasmid for expressing the SpCaVP1 protein was designated pFastBac-HSSc-SpCaVP1. The codon-optimized EDIII DNA fragment was synthesized (Genewiz, Suzhou, China) based on the sequence of Dengue virus 1 (GenBank No. KM204119). The EDIII sequence was then amplified using primers (1DIII-Fw: 5′-agttatgttatgtgcaccgg-3′; 1DIII-Rv: 5′-gcccaaaatagccattcgcc-3′) and further ligated into pFastBac-cSpyTag [24]. The DNA sequence also encoded poly-tags (an EDIII-cSpyTag-TEV-Strep-Flag, SpyTag, TEV protease cleavage site, Strep-Tag, and Flag-Tag). The resulting plasmid for expressing the EDIII protein was designated pFastBac-FSS-EDIII. Both plasmids were constructed and then utilized for the generation of a recombinant BmNPV bacmid. Subsequently, the recombinant baculovirus was generated in cultured Bm5 cells according to our previous reports [31]. The cell culture supernatant was collected and used for serial infections to obtain high-titer virus stocks, which were employed to infect silkworm larvae.

### Expression of recombinant proteins from silkworms

Fifth instar silkworm larvae (Ehime Sansyu, Ehime, Japan) were reared on an artificial diet (Silkmate S2, Nosan, Yokohama, Japan) in a rearing chamber (MLR-351H, Sanyo, Moriguchi, Japan) at 25 °C. On the second day, the 5th instar larvae were injected with 10 µl of a PBS solution containing 250 µl/ml recombinant baculovirus stock using a 1 ml syringe (26G × 1/2, 0.45 × 13 mm). Five days postinjection (dpi), the fat body was collected in 5 ml lysis buffer (0.2 mol/l Tris–HCl, pH 7.6, 0.1% IGEPAC, and protease inhibitor) from each silkworm. This solution was sonicated as follows: 10 s at an amplitude of 60–80 with 30 s on ice for each cycle, which was repeated 20 times. The sonicated solution was incubated on ice for 1 h and centrifuged at 4 °C and 8000×*g* for 15 min. The supernatant was filtered with a 0.2 µm filter and stored at − 80 °C until use.

### Lysis of recombinant protein expressed in E. coli (mCherry construct)

The *E. coli* cell pellet was resuspended in 3 ml ice-cold PBS for each 50 ml culture. To a 15 ml solution, 15 µl of 1 µg/ml lysozyme and 15 µl 1× complete Mini EDTA free Version protease inhibitor (from a 100 × stock solution, Roche, Tokyo, Japan) was added and incubated on ice for 30 min. Sonication was performed on the ice at an amplitude of 70 with a 30-s interval cycle for 20 min. This solution was then centrifuged at 12,000×*g* for 10 min at 4 °C. The supernatant was filtered with a 0.2 µm filter before further use.

### Preparation of Ni-conjugated magnetic nanoparticles (Ni-MNPs)

The superparamagnetic iron oxide nanoparticles (SPIONs) were synthesized by following Massart's method [32]. Five milliliters of ammonium hydroxide were added to 5 mmol of FeCl_2_ and 10 mmol of FeCl_3_ in 40 ml of ultrapure water. The mixed solution was vigorously stirred at room temperature for 30 min, and the synthesized MNPs were magnetically separated from the solution. Subsequently, the synthesized MNPs were coated with SiO_2_ for functionalization and stabilization [33]. The freshly synthesized SPIONs in 120 ml ethanol were sonicated at room temperature for 30 min, and 150 µl of tetraethyl orthosilicate (TEOS) was added to the solution. The MNP@SiO_2_ was separated and washed at room temperature after 6 h of stirring.

Amino group conjugation for MNP functionalization was performed by following previously reported protocols [34]. The washed MNP@SiO_2_ was dissolved in 100 ml anhydrous toluene, sonicated for 30 min, and then loaded into a three-necked round-bottom flask. (3-Aminopropyl)trimethoxysilane (APTMS) was added slowly and heated at 40 °C with vigorous stirring for 24 h. The product was magnetically separated and washed with ethanol several times.

Ni-modified MNP@SiO_2_@NH_2_ was prepared as previously reported [35]. Briefly, 0.32 g of isatoic anhydride was added to the solution, and 0.5 g of amino benzamide (2-AB)-immobilized MNP@SiO_2_@NH_2_ was dissolved in 100 ml of anhydrous toluene and refluxed for 12 h. The prepared MNP@SiO_2_@NH_2_@2-AB was separated by magnetic decantation and washed with ethanol several times. MNP@SiO_2_@NH_2_@2-AB (0.5 g) was suspended in 100 ml of ethanol and ultrasonically dispersed to form a homogeneous solution mixed with 2 mmol Ni(OAc)_2_·4H_2_O and refluxed for 12 h. The Magnetically separated MNP@SiO_2_@NH_2_@Ni was washed with ethanol to remove unreacted agents and dried overnight.

Each sample was characterized using transmission electron microscopy (TEM; JEM-2100F, JEOL, Ltd., Tokyo, Japan) with energy dispersive X-ray spectrometry (EDS) for elemental mapping nickel and iron. The zeta potential and hydrodynamic particle size were measured by dynamic light scattering (DLS) using a Zetasizer Nano series (Malvern Inst. Ltd., Malvern, UK).

### Strep-tag affinity chromatography

Purification of mCherry from the *E. coli* cell lysate was performed using the Strep-Tactin affinity column with a manual peristaltic pump at a low flow rate (0.5 ml/min). The processed lysate was filtered through a 0.8 µm filter before chromatography was performed. The elution fraction, which had a reddish/purple color and strong red fluorescence, was separately collected.

### General MNP purification protocol

Purification with the commercial magnetic bead MagneHis (Promega, Tokyo, Japan) was performed according to the manufacturer's manual, but the amounts used varied depending on the scale. Usually, the Ni particles were vortexed before usage, and then 100–300 µl of particles (2 mg/ml MNP solution) were added to a 1 ml sample. Incubation was performed for 10 min after mixing, but the mixture was stirred for 2 h at 4 °C for scaling up. For the magnetic separation a neodymium magnet (TRUSCO Nakayama, Tokyo, Japan) was used. The supernatant was removed, and 500 µl washing buffer (100 mmol/l HEPES and 10 mmol/l imidazole) was added and mixed. After magnetic separation, the previous step was repeated two times. For elution, 200 µl elution buffer (100 mmol/l HEPES and 500 mmol/l imidazole) was used. A different buffer (20 mmol/l Tris–HCl, 0.5 mol/l NaCl and 1 mol/l imidazole, pH 7.5) was applied to ensure complete elution.

For the MNPs prepared, as described in “2.5” section, the volume of sample/buffers/MNPs used varied in each protocol. In most cases, only two elution steps were performed. The basic sequence of the protocols was as follows. The MNPs were sonicated in a water bath for 30 min, and 350 µl of 2 mg/ml solution of MNPs were added to 250 µl of sample and 200 µl of washing buffer (20 mmol/l Tris–HCl, 0.5 mol/l NaCl and 20 mmol/l imidazole, pH 7.5). These were mixed and then incubated on ice for 30 min with occasional gentle mixing. After magnetic separation, the supernatant was removed, 200 µl washing buffer was added, and the mixture was incubated on ice for 10 min. After magnetic separation was performed 2 times, following buffers were used for elution: a weak elution buffer (20 mmol/l Tris–HCl, 0.5 mol/l NaCl and 300 mmol/l imidazole, pH 7.5), a strong elution buffer (20 mmol/l Tris–HCl, 0.5 mol/l NaCl, or 1 mol/l imidazole, pH 7.5). The amount depended on the protocol. The incubation time was 30 min on ice with occasional gentle mixing. After magnetic separation, the previous step was repeated with a strong elution buffer. For the volumes of the individual purification protocols, which are variable or not mentioned here, please see the figure legends which contain the specific information for the respecting figure. For all magnetic separations, the time to attract all MNPs were at least 5 min, as the case may also be longer, to ensure that all MNPs settled.

### Optimized MNP3 purification protocol

The MNPs were sonicated in a water bath for 30 min, and 2 ml of 3.75 mg/ml MNPs were added to 1 ml fat body sample and 600 µl washing buffer (20 mmol/l Tris–HCl, 0.5 mol/l NaCl, and 20 mmol/l imidazole, pH 7.5). These were mixed and then incubated on ice for 30 min with occasional additional mixing. After magnetic separation, the supernatant was removed, 200 µl washing buffer was added, and the mixture was incubated for 10 min on ice. After magnetic separation, the washing step was repeated. After magnetic separation was performed during the first elution step, 500 µl of weak elution buffer (20 mmol/l Tris–HCl, 0.5 mol/l NaCl, and 300 mmol/l imidazole, pH 7.5) was used. For the second elution step, 500 µl of strong elution buffer (20 mmol/l Tris–HCl, 0.5 mol/l NaCl, and 1 mol/l imidazole, pH 7.5) was used. As the third elution step, the second elution step was repeated. The incubation was performed for 30 min on ice with occasional additional mixing. For all magnetic separations, the time to attract all MNPs were at least 5 min, as the case may also be longer, to ensure that all MNPs settled.

### SDS-PAGE

The samples were investigated using 10% polyacrylamide gels. They were diluted with an equal amount of sample buffer (0.125 mol/L Tris–HCl, 4% SDS, 20% glycerol, 0.01% mercaptoethanol, and 0.15 mmol/L bromophenol blue), mixed, and heated at 95 °C for 5 min for denaturation. Electrophoresis was carried out with a BioRad SDS-PAGE chamber with a PowerPac Basic (BioRad, Hercules, CA, USA). The constant voltage was set at 90 V for the stacking gel and 120 V for the running gel. The size classification was performed with the PM1700 ExcelBand standard (Smobio, Hsinchu City, Taiwan). The 10% acrylamide gel was stained with Coomassie G-250 for approximately 2 h with a short heating period. Usually, the gels were destained with deionized water for at least 2 h to achieve adequate contrast. For documentation, the gels were scanned with the printer system Apeos Port IV (Fuji Xerox, Tokyo, Japan). The samples were analyzed with a series of dilutions from 8 µl sample to 22 µl sample in sample buffer. Each time, 15 µl of the dilution was loaded in the gel lane.

### Western blotting

For western blotting, the proteins were subjected to SDS-PAGE and transferred to polyvinylidene fluoride (PVDF) (Immobilon-P, Merck, Tokyo, Japan) membranes using the Trans-Blot SD Semi-Dry Transfer Cell (Bio-Rad, Tokyo, Japan). Blocking was performed for at least 1 h with 15 ml 5% skim milk in TBS containing 0.1% Tween 20. After washing with TBS and incubation for at least 2 h with mouse anti-Strep-tag antibody (1:10,000, QIAGEN, Tokyo, Japan), the blots were rewashed. Incubation with a secondary goat anti-mouse IgG-horseradish peroxidase (HRP) (1:10,000, MBL, Nagoya, Japan) was performed for at least 1 h. Immunoreactive bands were visualized using the Immobilon ECL Ultra Western HRP Substrate (Merck K. K., Tokyo, Japan) on the Versa-Doc 4000 MP (BioRad, Hercules, CA, USA).

## Results and discussion

### ***Characterization of Ni***^***2***+^***magnetic nanoparticles***

**Fig. 1:**
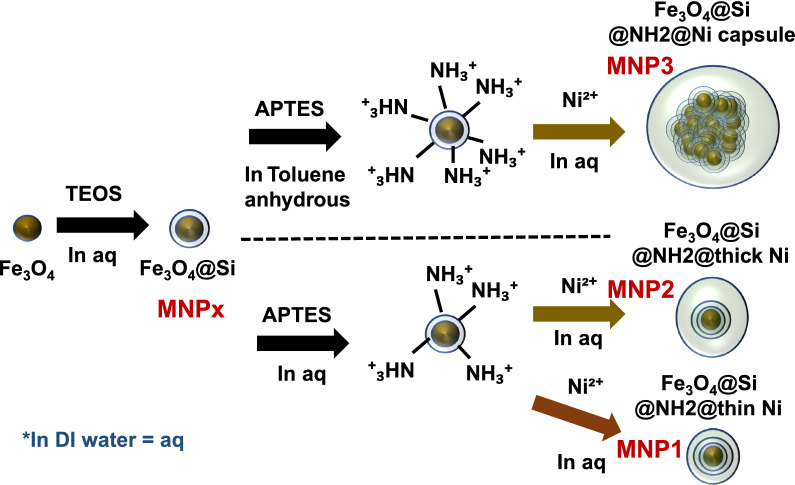
Three different nickel-modified magnetic nanoparticle synthesis procedures

In this study, 4 types of prepared MNPs (Fig. 1) were designated: MNPx, Fe_3_O_4_@SiO_2_, Fe_3_O_4_@SiO_2_@NH_2_@thin Ni; MNP1, Fe_3_O_4_@SiO_2_@NH_2_@thick Ni; MNP3, Fe_3_O_4_@SiO_2_@NH_2_@capsule Ni; MNP3.

**Fig. 2:**
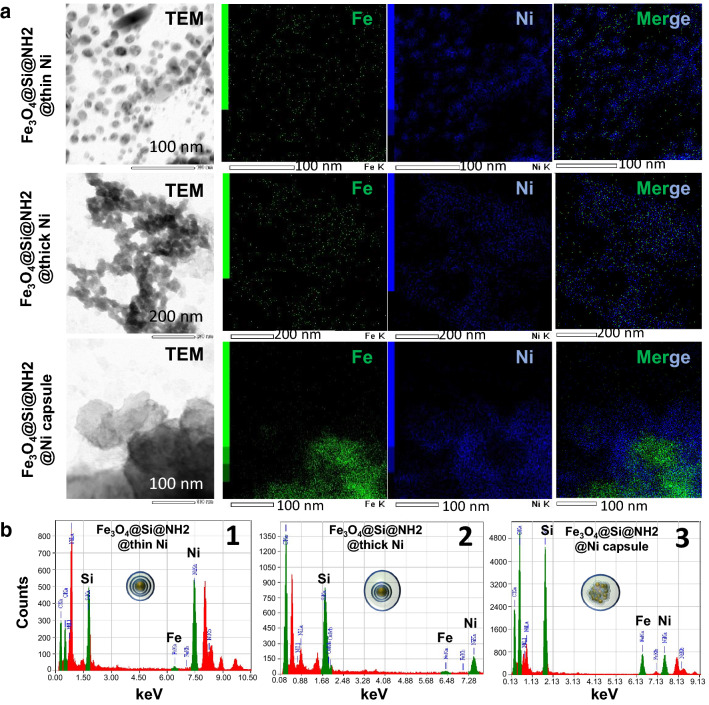
**a** Morphology of the three types of magnetic nanoparticles observed by TEM and EDS's elemental mapping results. **b** Elemental content analysis of each magnetic nanoparticle by EDS. B-1, 2, and 3 indicate MNP1, MNP2, and MNP3, respectively. The Si thin film was modified on the surface of the synthesized MNPs. An amino group was added to the surface of the SI thin film using APTES, and 2-amino benzamide (2-AB) and Ni(OAc)_2_ were reacted stepwise. Ni was chemically modified on the MNP surface prepared under each condition

The MNP surface zeta potential of each step up to the nickel coating was measured (Additional file 1: Fig. S1). The non-functionalized MNPs showed negative charges, and the silica coating and the nickel coating showed a clear positive surface zeta potential. This result clearly indicates the nickel coating on the MNP surface. The conjugation of MNPs by Ni was observed by TEM (Fig. 2a), and EDS was used to analyze each sample for elemental mapping. MNP3 had a capsule-like structure and formed larger particles compared with MNP1 and MNP2. This capsule structure contains many MNPs, as judged from Fe's green signals (Fig. 2a, Fe panel). The capsule structure significantly increased the number of MNPs per Ni particle, which was also proven by the elemental analysis results. In MNP1 and -2, the Fe content relative to the Ni content was small, and it was presumed that the magnetic force used for protein separation was not enough (1, 2 of Fig. 2b). Since MNP3 maintained a capsule shape, it contained balanced amounts of the element's Fe and Ni (3 of Fig. 2b). The advantage of MNP3 is that stable magnetic separation could be achieved without the attenuation of the magnetism when a large amount of protein adhered to the particle surface. The hydrodynamic size of the final products, MNP1, 2, and 3, was also measured by dynamic light scattering measurements (Additional file 1: Fig. S2). Each functionalized particle shows a different hydrodynamic size, consistent with the size of the particles observed by TEM.

**Fig. 3:**
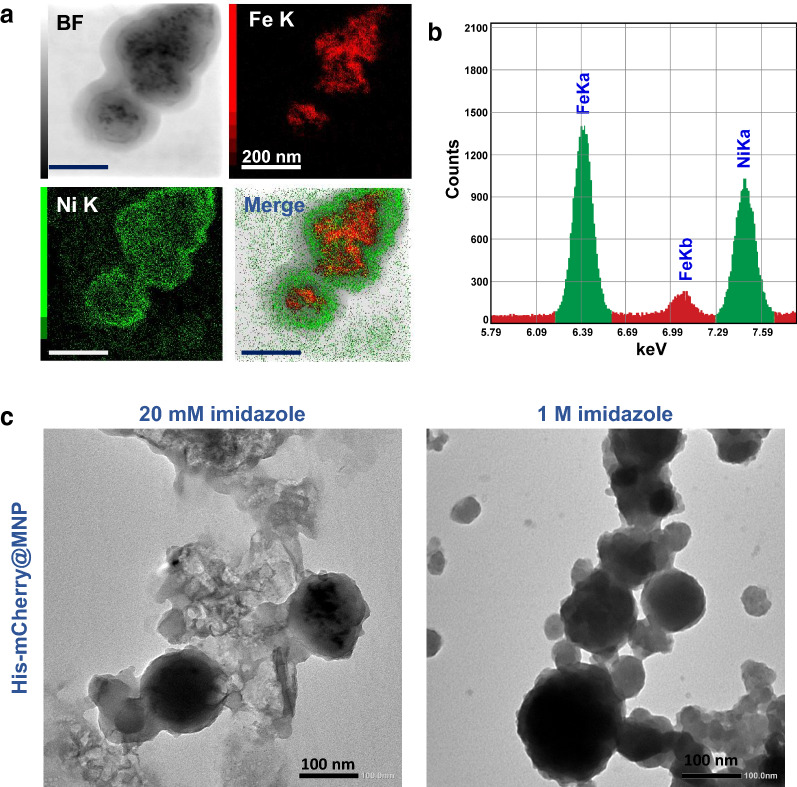
**a** TEM image of MNP3 and elemental mapping analysis. **b** Distribution of nickel and iron content in MNP3. **c** Images of MNP3 treated with different concentrations of imidazole after magnetic separation

MNP3 was analyzed in detail using HRTEM and high-sensitivity EDS (Fig. 3a, b). The capsule's size was approximately 100 nm to 200 nm that fit with the dynamic light scattering measurement result, and the thickness of the Ni capsule that formed to cover the MNP was approximately 20 nm. It was confirmed by EDS elemental mapping that a large amount of Fe was contained in most Ni capsules. Magnetic separation of MNP3 from the solution was also carried out using a magnet, and MNP3 was separated from the MNP3 solution at a concentration of 10 mg/ml in 10 s (Additional file 1: Fig. S3). The detailed analysis has shown that MNP3 has sufficient magnetic force as a nanomaterial for magnetic separation. The His-tagged protein is trapped by the formation of a complex with Ni on MNPs. When imidazole, which has a higher affinity for metals than histidine, is added, the metal forms a complex with imidazole, which causes the protein-metal complex to detach. This principle is commonly used for affinity purification, whereby low imidazole concentrations can even be used to increase the binding specificity [36]. The His-tagged protein targeted by MNP was treated under different imidazole concentration conditions, and the MNP surface was observed by TEM (Fig. 3c). It was suggested that many proteins remained adsorbed to the MNPs at an imidazole concentration of 20 mM. Since no protein was observed on the surface of MNPs at an imidazole concentration of 1 M, it was used as the optimum condition for subsequent experiments.

### Preliminary purification comparison tests

**Fig. 4:**
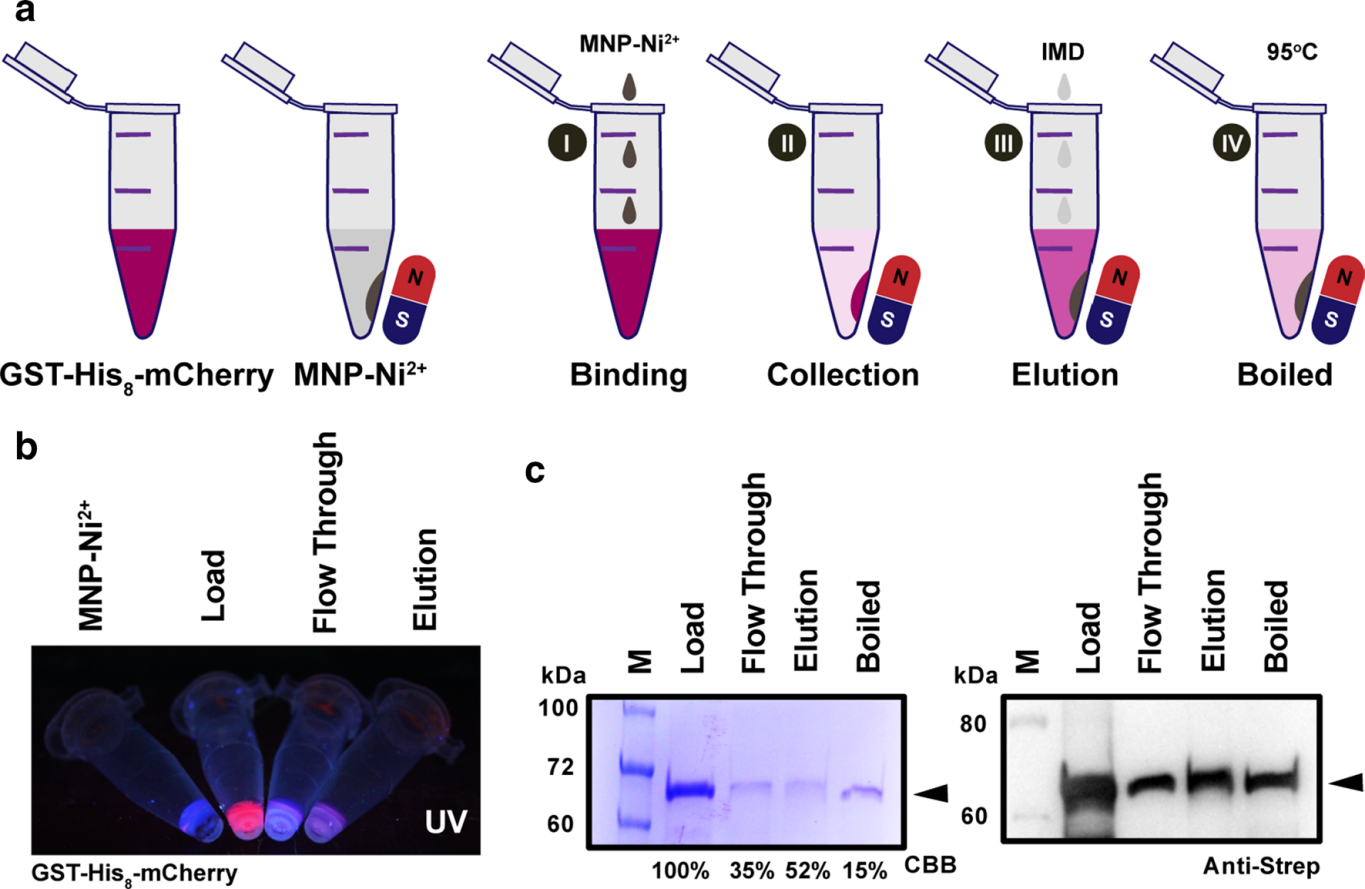
Preliminary binding test of the GST-His8-mCherry protein purified using MNP1. **a** Schematic diagram of protein separation using MNP1. A protein of interest such as GST-His_8_-mCherry (red) was employed for protein-MNP capture, and the released mCherry protein could be visualized by UV detection during the whole process. The study was simply performed by allowing the protein to bind (I) and to be collected by a magnet (II), followed by washing and elution (III). The remaining bound protein on the MNPs was denatured by heating (95 °C, 10 min) in SDS-PAGE loading buffer (IV). **b** Bare MNP1, loading, flow-through, and elution samples were illuminated under UV light. (C) MNP1 allowed efficient single-step purification as proven by SDS-PAGE (left) and western blot analysis using Anti-Strep-tag II (right)

In short, the process of using MNPs for purification involves adding the modified MNPs to the sample solution, which then binds to the target protein, and the complex is then magnetically separated from the surrounding matrix. The matrix is then removed, which is followed by washing and elution steps so that in the end, theoretically, the desired protein is released from the MNPs and is the only protein in the solution (Fig. 4a). MNP1 was then tested using a purified recombinant fluorescent mCherry protein, which can be easily visualized as pink-colored under UV light. As demonstrated in Fig. 4b, the flow-through had a decreased fluorescence intensity as visualized under UV, indicating that the MNPs were successfully modified with Ni and showed active binding of His-tagged proteins. Moreover, mCherry signals were also detected from the eluted samples when 500 mM imidazole was used to elute the protein from MNP1 with an approximately 15% purification recovery rate calculated based on the loading amount. Taken together with the SDS-PAGE and western blotting results (Fig. 4c), these results led us to conclude that the prepared MNP1 is usable for purification of His-tagged proteins.

To compare the MNPs prepared in this study, MagneHis magnetic beads and MNP1 were used to purify the SpCaVP1 + EDIII protein from the silkworm fat body. To avoid a high degree of unspecific binding of proteins to the magnetic particles, a low concentration of imidazole was added to the washing buffer: 20 mmol/l for the particles prepared in this work, and 10 mmol/l for MagneHis as recommended by the manufacturer. Three hundred mmol/l was used for the MagneHis magnetic beads and 500 mmol/l imidazole for our MNPs in the first elution buffer, and the second elution buffer contained 1 mol/l imidazole in all cases. The SpyTag/-Catcher-linked coexpressed protein has approximately 95 kDa, and MNP1 has a low binding affinity for the His-tagged protein in crude protein samples. The MagneHis magnetic beads, in contrast, were able to bind the target protein and to separate it from other proteins, mainly in the first elution fraction (Additional file 1: Fig. S4). Together with our targets, most impurities were eluted from the magnetic beads by 1 mol/l imidazole. However, the purified proteins were still at a very low concentration. They were not visible after Coomassie staining (Additional file 1: Fig. S4), even when the MagneHis MNPs were used on a large purification scale (Additional file 1: Fig. S5). The reason was not further investigated; one main reason could be that the magnetic beads' loading capacity is with 5 µg Ubiquitin/mg MNP not very high. Incomplete elution could be ruled out, as shown in Additional file 1: Fig. S4.

The above negative results raised whether the protein sample from the silkworm fat body as a sample matrix is too difficult to purify for the proof-of-concept. Therefore, purification was then repeated with the *E. coli* cell lysate containing a His-tagged mCherry protein as a model sample. One additional benefit of using mCherry is the intense red fluorescence (Ex. 540–590 nm, Em. 550–650 nm) and the red color, which makes the protein's tracking during the purification process easier. Using this sample matrix and model protein, MNP1 and MNP2 were able to bind and separate mCherry from the host cell proteins (Additional file 1: Fig. S6A–B). MNP2 was more effective than MNP1 because of the reduced nonspecific binding of the target protein to the MNP itself, as it could not be eluted from the MNP (Additional file 1: Fig. S6B). Moreover, the protein amount in the MNP2 elution fraction was higher than that in the MNP1 elution fraction (Additional file 1: Fig. S6B), which was also observable by fluorescence, as the red fluorescence of elution fraction 1 (E1) from MNP2 was much stronger than that from MNP1 (Additional file 1: Fig. S6C).

### Ni-modified highly dispersible MNPs

**Fig. 5:**
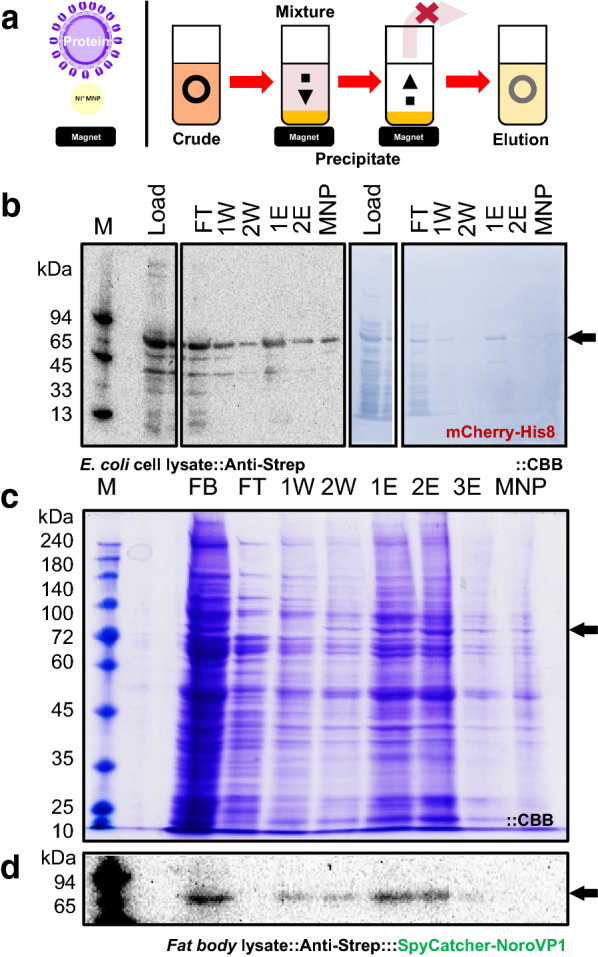
Purification of proteins from the fat body of silkworms with MNP3. **a** Principle of MNP purification. MNPs bind to target proteins, both are separated from impurities via a magnet, and the supernatant is discarded. After removing the magnet, the target protein-bound MNPs are resuspended (e.g., in elution buffer). **b** Western blot and CBB-stained membrane of His-tagged mCherry protein purified using MNP3. The elution was performed with 50 µl of 1 mol/l imidazole. The sample was diluted in a volume of 8 µl to 22 µl, and in each lane, 15 µl was loaded. **c** Optimized and scaled-up (1 ml sample) purification of proteins from the fat body of the silkworm with MNP3 shown by Coomassie blue-stained SDS-PAGE (**c**) and western blotting results (**d**) of VP1 purification using MNP3. For both, the washing buffer contained 20 mmol/l imidazole, with 1 ml fat body and 7.5 mg MNPs. The first elution was performed with 300 mmol/l, and the second and third elution was performed with 1 mol/l imidazole (500 µl each). An 8 µl to 22 µl dilution was performed, and 15 µl was loaded in each lane FB: Fat body; FT: Flow-through; W1: 1st Washing fraction; W2: 2nd Washing fraction; E1: 1st Elution fraction; E2: 2nd Elution fraction; MNP: MNPs; M: Marker; Black arrow indicates the target protein

During the purification process, illustrated in Fig. 5a, the previously prepared MNPs tended to aggregate easily and stick to the tube walls during the purification process. This complicated the whole purification process and increased protein loss, which reduced the recovery of the target proteins. Our magnetic nanoparticles were further improved to be more easily dispersed in aqueous solutions to tackle these problems. MNP3 was then also tested for use in purification because of its high dispersibility. The purification result from E. coli extract is shown in Fig. 5b. Using MNP3 for comparison, the previous mCherry purification process was repeated, during which the sample amount was slightly increased from 250 to 300 µl, and the MNP amount was somewhat reduced from 350 to 300 µl of a 2 mg/ml MNP solution. The purification results for MNP3 were better than those for MNP2. Not only was nonspecific binding decreased significantly, but the amount of the eluted target protein was also increased compared to that eluted from MNP2, and the specificity of the eluted proteins improved, as shown by the western blotting results (Additional file 1: Fig. S7A–C). The decrease in nonspecific binding and the improved elution ability were also visible on the CBB-stained western blotting membrane; therefore, on the latter membrane, a strong band for the target protein mCherry was visible (Additional file 1: Fig. S7A). On the other hand, the fluorescence assay and western blotting results show that compared to binding to MNP2, a large amount of the target protein could not bind strongly enough to MNP3 and was present in the washing fractions (Additional file 1: Fig. S7B–C). This is also supported by the samples' fluorescence emission obtained from the different purification steps (Additional file 1: Fig. S7C).

### Purification of protein from a complex sample matrix using MNP3

After proving the functionality of MNP3, the purification of His-tagged SpCaVP1 + EDIII was performed from the silkworm fat body. The protocol was similar to that used for MNP1 and MNP2. The significant changes were the increase in the MNP amount (from 100 µl to 500 µl 2 mg/ml solution) and 1 mol/l imidazole instead of only 300 mmol/l the first elution. This time, the recombinant protein was separated successfully with the MNP3, and most target proteins were already eluted in the first step so that only a small loss occurred due to strong binding to the MNPs (Additional file 1: Fig. S8), which was also assessed by comparison to the results obtained using mCherry as the sample (Additional file 1: Fig. S7). On the other hand, using 1 mol/l imidazole directly as the first elution buffer proved to be disadvantageous. Even if the western blot band corresponding to the target protein was intense, the band could not be clearly distinguished from that of the eluted nonspecifically bound host cell proteins. This is different from the previous result, where the target protein comprised the main protein band, and few impurities were detectable (Additional file 1: Fig. S7A).

Nevertheless, the efficiency of MNP3 for the purification of recombinant proteins from a complex matrix was proven, as a high protein recovery could be achieved. Especially as we already showed in other studies that high purity and high recovery by simultaneous high host cell reduction from the silkworm system is very challenging so far [12, 13]. On the other hand, the purification is promising, but not satisfying enough.

Therefore, this protocol was scaled up with a SpCaVP1 sample amount of 1 ml fat body, 4.6 mg MNPs, 300 mmol/l imidazole as the first elution step, and the last two elution's were performed with 1 mol/l imidazole. The elution was performed with a volume of 500 µl, which led to the concentration of the target protein VP1. However, the loss of VP1 increased significantly during the washing steps, which consisted of 500 µl 20 mmol/l imidazole (Additional file 1: Fig. S9). To tackle this issue, the protocol was changed by removing imidazole from the washing buffer but not from the buffer used for the initial incubation and by increasing the MNP amount. This resulted in high VP1 recovery by reducing loss via washing and direct binding to the MNPs. As a result, the nonspecific binding to the MNPs increased (Additional file 1: Fig. S10), which was greater than expected. As the recovery also significantly improved, it was hypothesized that both effects were affected by the MNP amount.

To test this and decrease the unspecific binding, the protocol was again optimized by reintroducing 20 mmol/l imidazole to the washing buffer and increasing the MNP amount to 7.5 mg. This resulted in a similar high recovery of VP1 but also in a surprisingly high level of nonspecific binding to the MNP3s, even if it was slightly better than the previous (Fig. 5c, d). This leads to the hypothesis that proteins are easily bound to MNPs because of the large amount of histidine residues in proteins and that proteins from the silkworm fat body have a strong affinity for MNPs. However, proteins from the *E. coli* cell extract did not show this kind of behavior, as previously described (Additional file 1: Fig. S7A). It is a known fact that other proteins as the target proteins show affinity to the separation matrix, especially from eucaryotic systems, and His-tag affinity protocols take this into account [36]. Based on the imaging software results, by using the supposed target protein bands from the CBB staining and protein assay, we estimated roughly that we were able to reduce the protein amount in elution fractions 1 and 2 by approximately 77.7%, and the recovery ratio of VP1 was estimated to be 50.8%. This result is not a completely perfect purification as it is usually desired: One step, no target protein lost, and high purity. However, this is a rare result in reality. Our result is an additional useful method as a pretreatment before further chromatography steps to significantly reduce the host protein amount. This is especially interesting, as already shown. First, good separation and purity with our target protein from the *E. coli* extract were achieved (Fig. 5b), and second, a good protein reduction from the silkworm fat body matrix was achieved. The latter one is very intriguing, as the purification from this eucaryotic system is still troublesome and a big limitation for this system's usage. We reviewed the problems of the silkworm purification already elsewhere [7] and also showed that the purification is indeed not easy [12, 13]. Therefore, we will not discuss this aspect exhaustively any further. Another aspect that has to be mentioned is that the purification protocol still has room for further optimization as shown with our results, especially the time aspect, but this was not inside the scope of this present work and is under ongoing investigations.

Furthermore, the binding behavior of the MNP3s were investigated using mCherry as a reference and BSA as a negative control, which should not be able to bind specifically to the MNPs. With TEM, sample incubation was investigated with MNP3s after the magnetic separation and removal of the supernatant. After the two washing steps, the elution solution contained MNPs, 1 mol/l imidazole buffer, and in some cases, the sample (Additional file 1: Fig. S11). These images show that mCherry is explicitly bound to the MNPs and was released by the elution buffer (Additional file 1: Fig. S11A–B). However, it is also clear that BSA has a high nonspecific affinity for the MNPs, clustered around the MNP3s. Nevertheless, it was removed later after the elution step, as only a small amount was visible in the TEM images after elution (Additional file 1: Fig. S11C–D). This supports the conclusion that our MNP3s are a promising tool for pretreatment to reduce impurities before further purification procedures.

### ***Binding study of MNP3 with a highly efficient NH***_***2***_*** coating***

Using mCherry prepurified via the Strep-tag affinity column, we intended to perform a binding study with the MNP3s. For this, a purified mCherry sample (120 µg/ml protein) with several different volumes was added to achieve a different loading amount (12 µg; 30 µg; 48 µg; 60 µg; 72 µg; 84 µg; 96 µg; or 108 µg) in 300 µl MNP3 solution (2 mg/ml), and the buffer volume was adjusted to 950 µl or 1.2 ml each time. Except for the prepurified mCherry sample with 120 µg protein/ml, the protein concentration could not be determined for any other sample with the Pierce BCA Protein Assay Kit (Thermo Scientific, Waltham, USA) or the BioRad Protein Assay (BioRad, Hercules, CA, USA) because of the interaction with imidazole and the very low protein concentration. Moreover, the small sample amount made it impossible to dialyze the sample after elution. Therefore, we tried to solve this issue using the intensity of the western blot bands as a reference (Additional file 1: Fig. S12). Unfortunately, the western blot bands were too unstable and inconsistent to be analyzed and calculated with the imaging software.

Nevertheless, these results already indicated several things. With an increase in the loaded protein amount, the loss in the flow-through fractions remained mostly the same, but the amount that could be eluted or was still bound to the MNPs increased significantly (Additional file 1: Fig. S12A). Protein was still bound to the MNPs, despite using a strong elution buffer with 1 mol/l imidazole. This raises two possibilities. Elution of the nickel MNPs is imidazole-dependent and concentration-dependent in terms of the already eluted amount of protein in the surrounding buffer. The other possibility is that the amount of bound target protein is very high. The relative amount of imidazole in the elution buffer used is too low to compete with all binding sites, despite the concentration of 1 mol/l. Therefore, with the highest amount of loaded protein, the second elution fraction also contained a high amount of target protein, which was not the case for the lower amount (Additional file 1: Fig. S12A). In the subsequent experiments, when more target protein was loaded, more of the protein was recoverable. The highest minimum binding capacity was 48.8 µg/mg MNP, and the minimum elution capability was 26.43 µg/mg MNP (data not shown). However, using imaging software to assess the CBB-stained protein bands for the elution (Additional file 1: Fig. S12B) and MNP fractions proved to be unreliable, and therefore, a qualitative approach was undertaken.

**Fig. 6:**
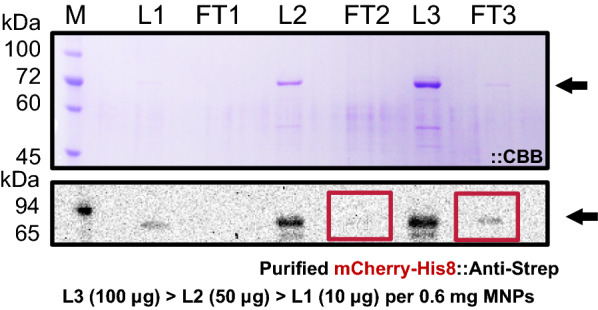
Binding study of the MNP3s. The capability of 0.6 mg MNPs to bind 10 µg, 50 µg, or 100 µg mCherry. Coomassie blue staining and western blotting results. FT: Flow-through; W1: 1st Washing fraction; W2: 2nd Washing fraction; E1: 1st Elution fraction; E2: 2nd Elution fraction; MNP: MNPs; L1–3: Prepurified loaded samples with the same target protein concentration but different amounts; M: Marker; black arrows indicate the target protein; red boxes indicate the corresponding western blot bands

By loading three different amounts of purified mCherry (10 µg; 50 µg; and 100 µg) and analyzing the western blotting results for the flow-through revealed that 0.6 mg MNP3s could bind at a minimum approximately 50 µg mCherry (Fig. 6). This is the binding capacity of approximately 83.3 µg/mg (50 µg/0.6 mg) MNP. The result also indicates that the binding capacity maybe even higher (little lower than 100 µg/0.6 mg = 166.7 µg/mg MNP), as the western blotting results for FT3 were not considerably better than those for FT2 (Fig. 6). This could be due to the higher binding of the target protein at a higher concentration. Therefore, we showed that our MNP has a higher binding capacity than the commercial MagneHis particles used. A minimum binding capacity of approximately 83.3 µg/mg MNP, a release of approximately 50%, and with VP1, a recovery of about 50.8%; based on the company analysis certificate, the binding capacity of the commercial MagneHis is 5 µg Ubiquitin/mg MNP with a minimum release of 50%.

Furthermore, ubiquitin's molecular mass is approximately 8.6 kDa, and that of mCherry is 72.2 kDa. If we assume that the size is correlated with the mass, we can say that our model protein larger than ubiquitin. Although it is impossible to give an exact quantitative comparison, our binding capacity is an eightfold higher than that of MagneHis.

## Conclusion and outlook

A successful purification method was developed for His-tagged proteins using our prepared MNPs. Moreover, these MNPs can be easily synthesized and can purify the target protein from complex sample matrices. In terms of recovery and binding capacity, these MNPs had a higher capacity than the commercial MagneHis that used for comparison. However, this was only valid for one of the three different MNPs, which was further investigated. The most efficient type was MNP3, which has a size of 100 nm to 200 nm and has an approximately 20 nm-thick nickel covering. By EDS elemental mapping, a large amount of Fe inside the MNP3s was confirmed, which provided their magneticity. Using the MNP3s, the total protein amount in the elution fractions was reduced up to at least approximately 77.7% with a target protein recovery of 50.8% from the silkworm fat body. The fat body is a complex sample matrix. It contains a large amount of proteins and many different kinds of proteins and lipids, which cannot be easily separated during prior treatment. However, with our MNP3s, we could highly reduce the amount of impurities from the silkworm fat body matrix. Using a pretreatment step should improve the overall purity and recovery of recombinant proteins from the silkworm. The binding capacity of pure mCherry could be estimated to be at least 83.3 µg protein/mg MNP as the minimum binding capacity, with a target protein recovery of approximately 50.8%. The protein used to determine the binding capacity has an approximately eightfold higher mass, indicating that the MNPs have an even higher binding capacity than the commercial particles. We conclude that MNP3 is a suitable candidate for further investigations and simple purifications in laboratories, especially as a pretreatment step for complex sample matrices, such as the silkworm expression system.

## Additional file

**Additional file 1.** Additional figures S1–S12.

## Data Availability

All relevant data generated or analyzed during this study are included in this published article and its Additional file 1. The missing datasets used and/or analyzed during the current study are available from the corresponding author upon reasonable request.

## Additional file 1

**Additional file 1** accompanies this paper at 10.1186/s12951-020-00715-1.
