# Supplementary material for: Ni-modified magnetic nanoparticles for affinity purification of His-tagged proteins from the complex matrix of the silkworm fat body
Source: J Nanobiotechnology. 2020 Nov 6;18:159. doi: 10.1186/s12951-020-00715-1 (PMC7648358; doi:10.1186/s12951-020-00715-1)
Supplement: Supplementary file 1 — Additional file 1. Additional figures S1–S12. [file 12951_2020_715_MOESM1_ESM.docx]

## **Additional file**

# **Ni-modified magnetic** **nanoparticles for affinity purification of His-tagged proteins from the complex matrix of the silkworm fat body**

Short running title: Purification of **His-tagged protein** using highly dispersible MNPs

Robert Minkner^1,2,†^, Jian Xu^3,†,#^, Kenshin Takemura^1,†^, Jirayu Boonyakida^1^, Hermann Wätzig^2^, Enoch Y. Park^1,3,^^[[1]](#footnote-1)^

^1^ Department of Bioscience, Graduate School of Science and Technology, Shizuoka University, 836 Ohya, Suruga-ku, Shizuoka 422-8529, Japan

^2^ Institute of Medicinal and Pharmaceutical Chemistry, TU Braunschweig, Beethovenstr. 55, 38106 Braunschweig, Germany

^3^ Laboratory of Biotechnology, Green Chemistry Research Division, Research Institute of Green Science and Technology, Shizuoka University, 836 Ohya, Shizuoka 422-8529, Japan

E-mail:

robert.minkner@tu-bs.de (RM)

takemura.kenshin.16@shizuoka.ac.jp (KT)

xujian@sei.ecnu.edu.cn (JX)

jirayu.boonyakida.17@shizuoka.ac.jp (JB)

h.waetzig@tu-bs.de (HW)

park.enoch@shizuoka.ac.jp (EYP)

**Figure S1. Surface zeta potential of MNPs.** Fe₃O₄ bare MNP (Blue), silica-coated MNP (Red), and MNP3 (Green).


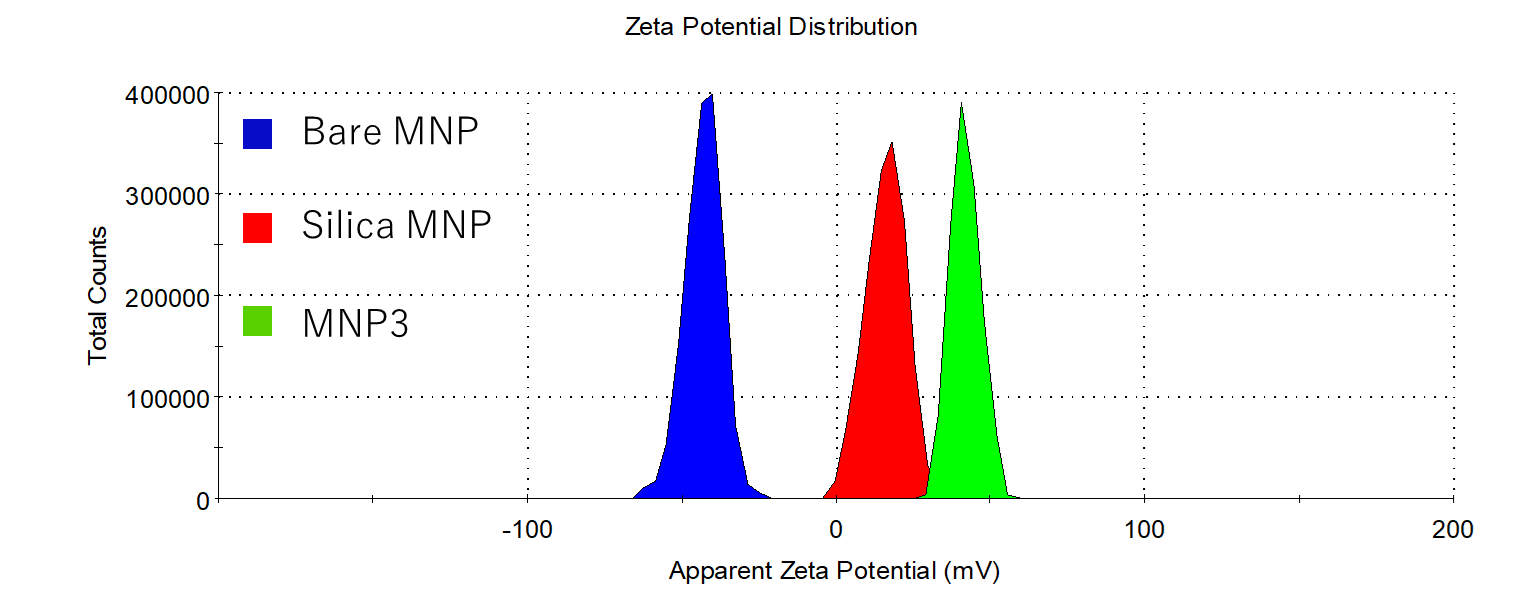


**Figure S2. Size distribution of MNPs using DLS.** MNP1 (Orange line), 2 (Red line), and 3 (Blue line).

**Figure S3. Magnetic separation of MNP3 using magnet.**


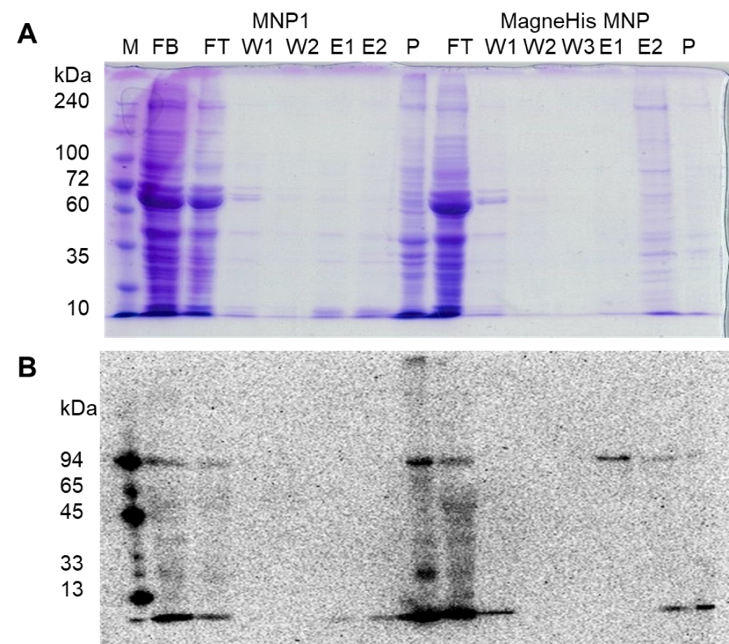


**Figure S4. Purification of SpCaVP1 + ED III fat body with self-made MNP1 and commercial beads. (A)** Coomassie blue staining SDS-gel and **(B)** Western Blot for the purification with self-made MNP1 and the commercial magnetic beads. Wash buffer for MNP1 contained 20 mmol/l imidazole and for the commercial beads 10 mmol/l. The first elution for the MNP1 was done with 300 mmol/l and the 2^nd^ with 1 mol/l imidazole. For the commercial beads it was 500 mmol/l and 1 mol/l, respectively. It was 8 µl sample to 22 µl dilution and on each line 15 µl was loaded. FB: fat body; FT: Flow through; W1: 1st Wash fraction; W2: 2nd Wash fraction; W3: 3rd Wash fraction; E1: 1st Elution fraction; E2: 2nd Elution fraction; P: MNPs; M is Marker.


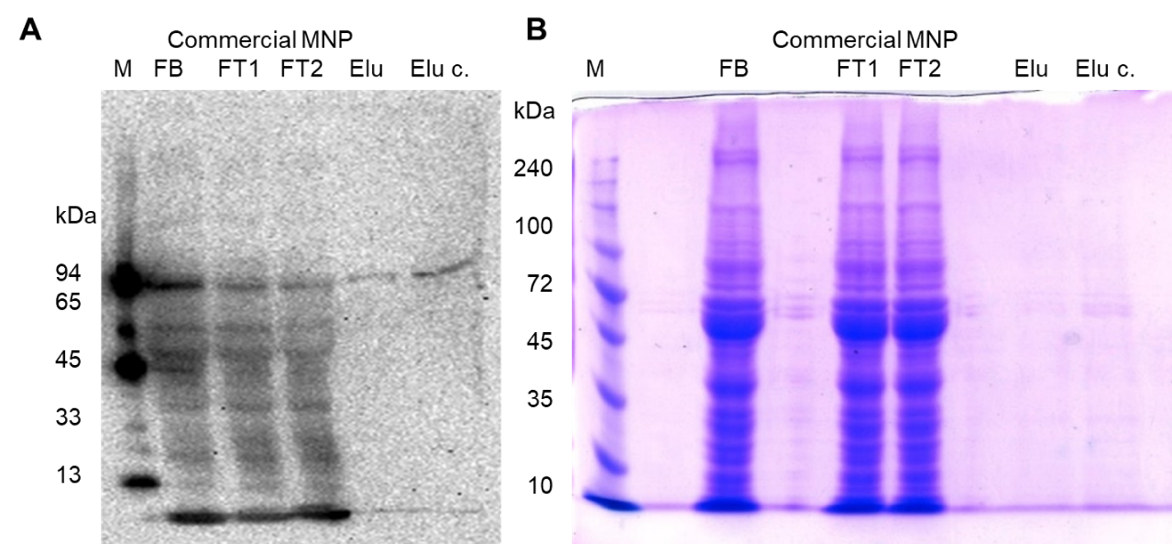


**Figure S5: Scaled up purification of SpCaVP1 + ED III fat body with commercial beads. (A)** Coomassie blue staining SDS-gel and **(B)** Western Blot for the purification with commercial magnetic beads. 3.4 ml sample and 400 µl magnetic beads were used. Wash buffer contained 10 mmol/l imidazole. The first elution for the magnetic beads was done with 500 mmol/l and the 2^nd^ with 1 mol/l imidazole, , each 800 µl. The 1^st^ flow through was repeatedly purified with regenerated magnetic beads and all elution fractions were pooled together. 8 µl sample to 22 µl dilution and on each line 15 µl was loaded. FB: fat body; FT1: Flow through after the first purification; FT2: Flow through from FT1 after repeatedly purification with regenerated magnetic beads; Elu: pooled elution fractions; Elu c.: Pooled and roughly threefold concentrated elution fractions; M is Marker.


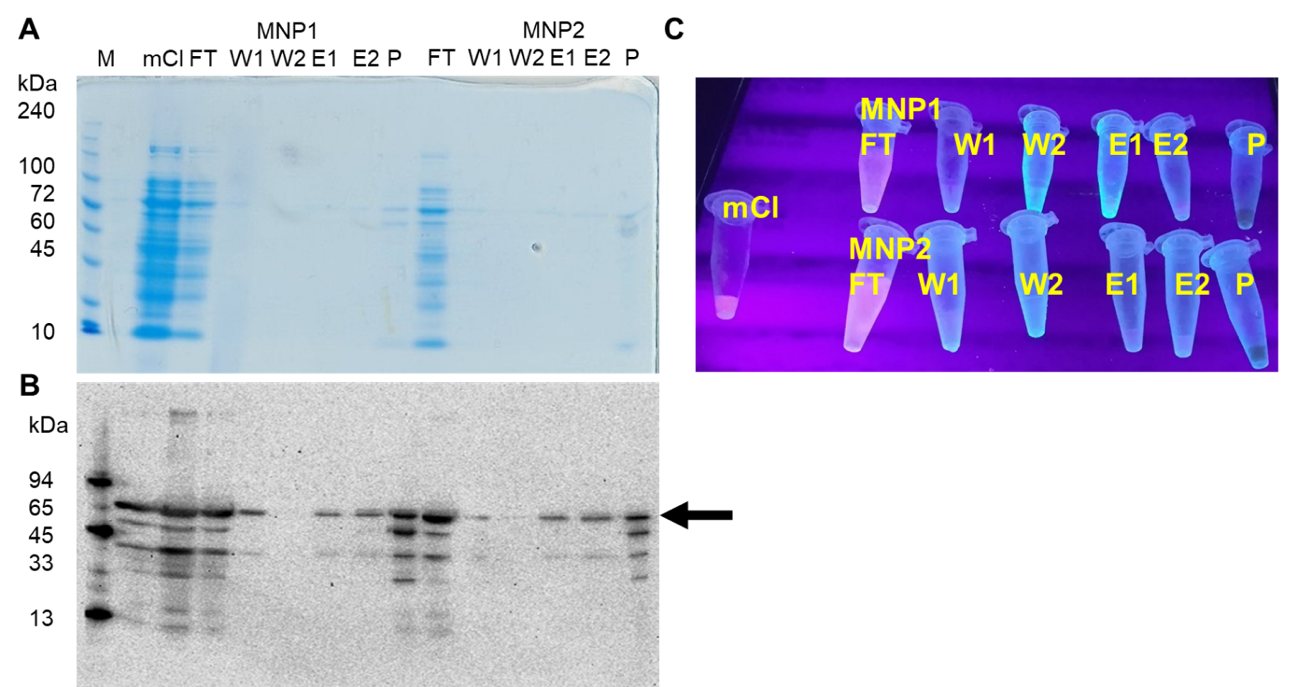


**Figure S6. Purification of mCherry *E. coli* cell lysate with self-made MNP1 and MNP2. (A)** Coomassie blue staining SDS-gel and **(B)** Western Blot for the purification with self-made MNP1 and MNP2. Wash buffer contained 20 mmol/l imidazole. The first elution was done with 300 mmol/l and the 2^nd^ with 1 mol/l imidazole, each 50 µl. It was 8 µl sample to 22 µl dilution and on each line 15 µl was loaded. **(C)** Fluorescence of the fractions. mCl: mCherry *E. coli* cell lysate; FT: Flow through; W1: 1st Wash fraction; W2: 2nd Wash fraction; E1: 1st Elution fraction; E2: 2nd Elution fraction; P: MNPs; M is Marker; Black arrow indicates target protein.


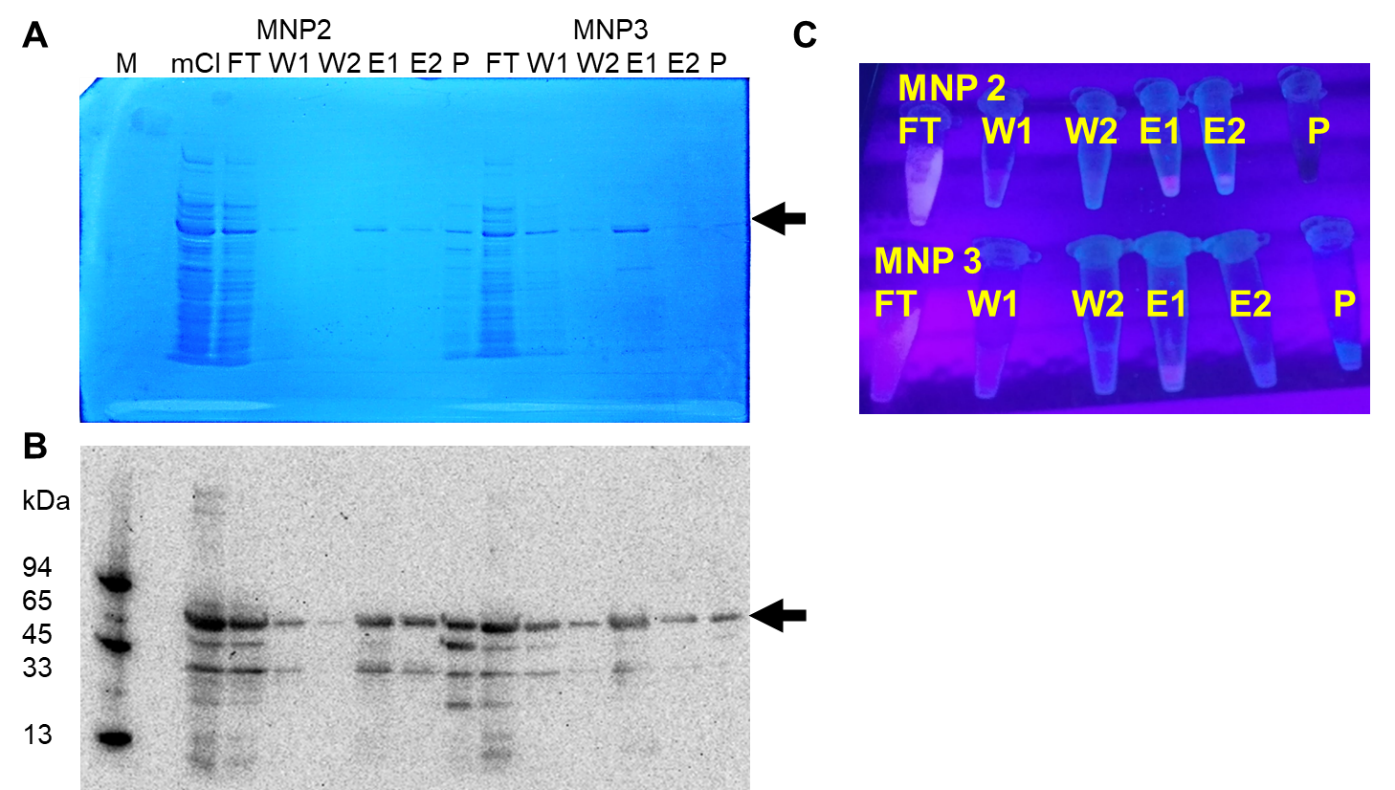


**Figure S7. Purification of mCherry *E. coli* cell lysate with self-made MNP2 and MNP3. (A)** Coomassie blue stained Western blot membrane and **(B)** Western Blot for the purification with self-made MNP2 and MNP3. Wash buffer contained 20 mmol/l imidazole. The elution for was done with 1 mol/l imidazole, each 50 µl. It was 8 µl sample to 22 µl dilution and on each line 15 µl was loaded. **(C)** Fluorescence of the fractions. mCl: mCherry *E. coli* cell lysate; FT: Flow through; W1: 1st Wash fraction; W2: 2nd Wash fraction; E1: 1st Elution fraction; E2: 2nd Elution fraction; P: MNPs; M is Marker; Black arrow indicates target protein


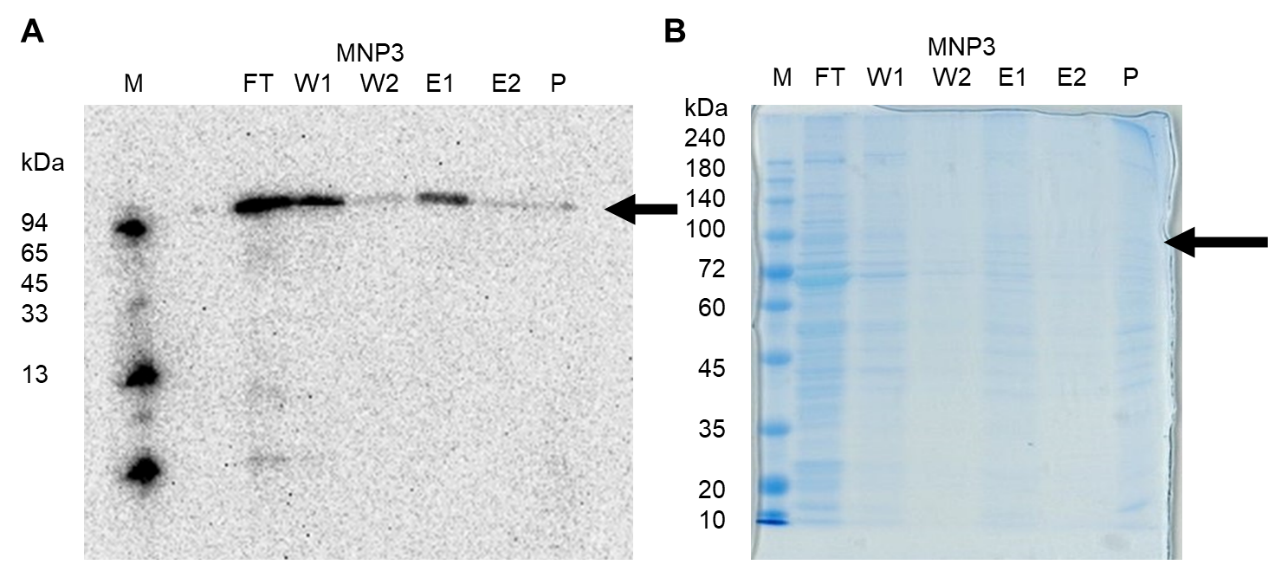


**Figure S8. Purification of SpCaVP1 + ED III fat body with self-made MNP3. (A)** Western blot and **(B)** Coomassie blue stained SDS-PAGE of the purification with self-made MNP3. Wash buffer contained 20 mmol/l imidazole. The elution for was done with 1 mol/l imidazole, each 50 µl. It was 8 µl sample to 22 µl dilution and on each line 15 µl was loaded. FT: Flow through; W1: 1st Wash fraction; W2: 2nd Wash fraction; E1: 1st Elution fraction; E2: 2nd Elution fraction; P: MNPs; M is Marker; Black arrow indicates target protein


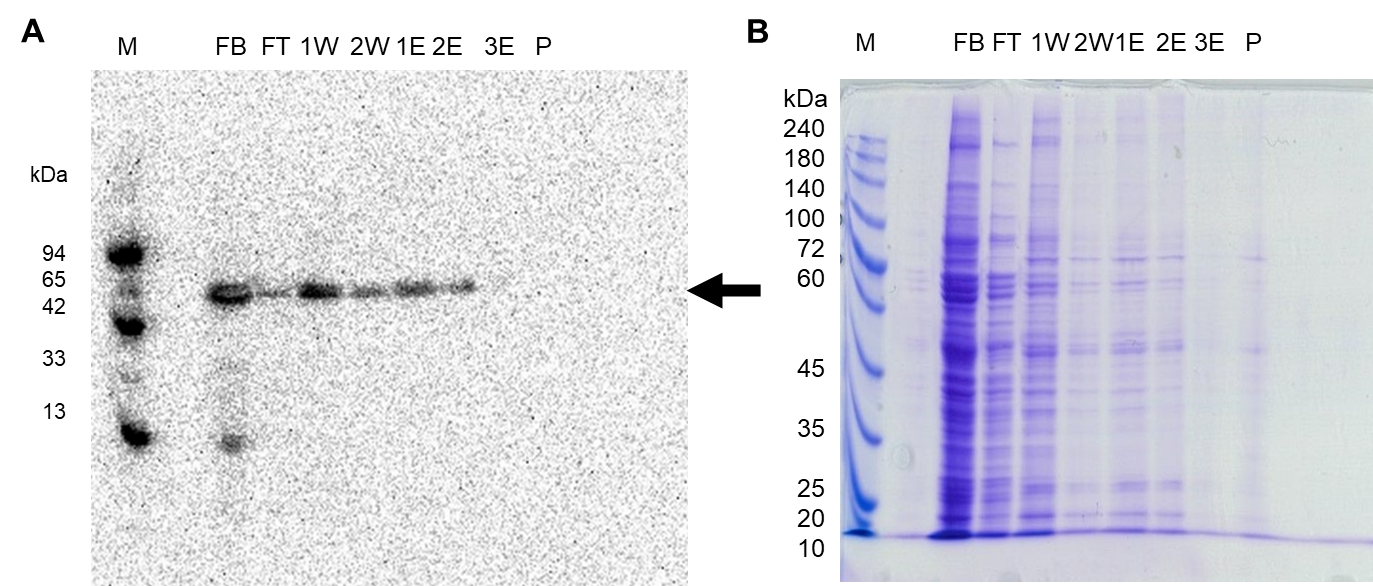


**Figure S9. Up-scaled purification of 1 ml SpCaVP1 fat body with self-made MNP3. (A)** Western blot and **(B)** Coomassie blue stained SDS-PAGE of the purification with self-made MNP3. Wash buffer contained 20 mmol/l imidazole and 500 µl were used. 1 ml fat body and 4.6 mg MNPs. First elution for was done with 300 mmol/l, the second and third with 1 mol/l imidazole, each 500 µl. It was 8 µl sample to 22 µl dilution and on each line 15 µl was loaded. FT: Flow through; W1: 1st Wash fraction; W2: 2nd Wash fraction; E1: 1st Elution fraction; E2: 2nd Elution fraction; E3: 3rd Elution fraction; P: MNPs; M is Marker; Black arrow indicates target protein


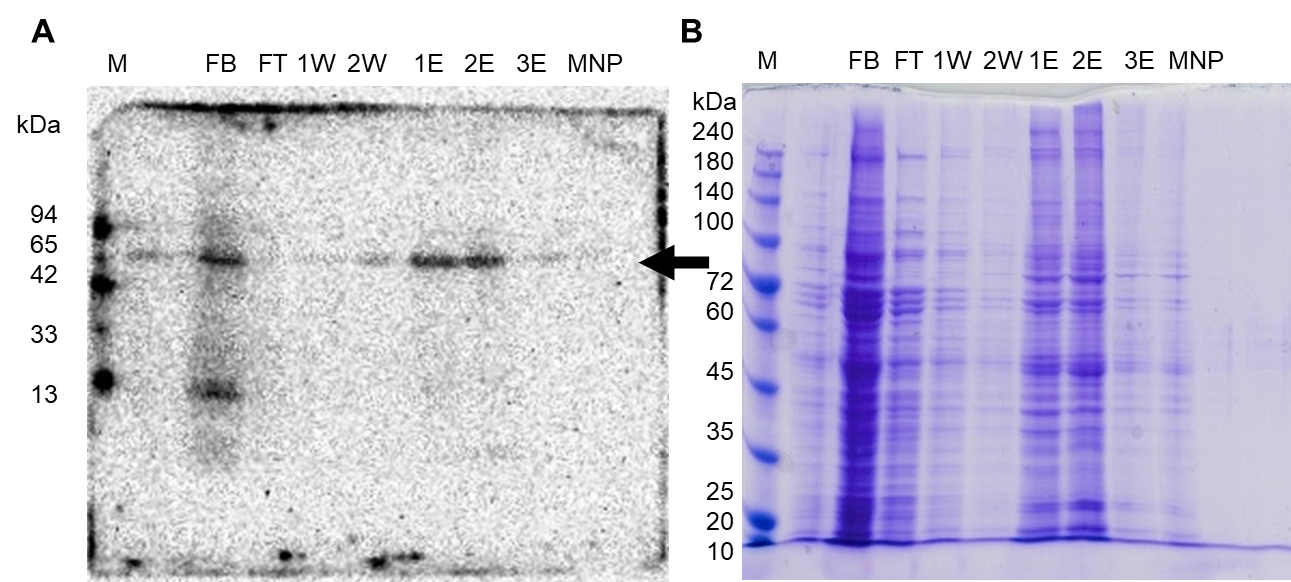


**Figure S10. Up-scaled purification of 1 ml SpCaVP1 fat body with self-made MNP3 without imidazole in wash buffer and more MNPs. (A)** Western blot and **(B)** Coomassie blue stained SDS-PAGE of the purification with self-made MNP3. Wash buffer contained no imidazole. 1 ml fat body and 5.7 mg MNPs. First elution for was done with 300 mmol/l, the second and third with 1 mol/l imidazole, each 500 µl. It was 8 µl sample to 22 µl dilution and on each line 15 µl was loaded. FT: Flow through; W1: 1st Wash fraction; W2: 2nd Wash fraction; E1: 1st Elution fraction; E2: 2nd Elution fraction; E3: 3rd Elution fraction; P: MNPs; M is Marker; Black arrow indicates target protein.


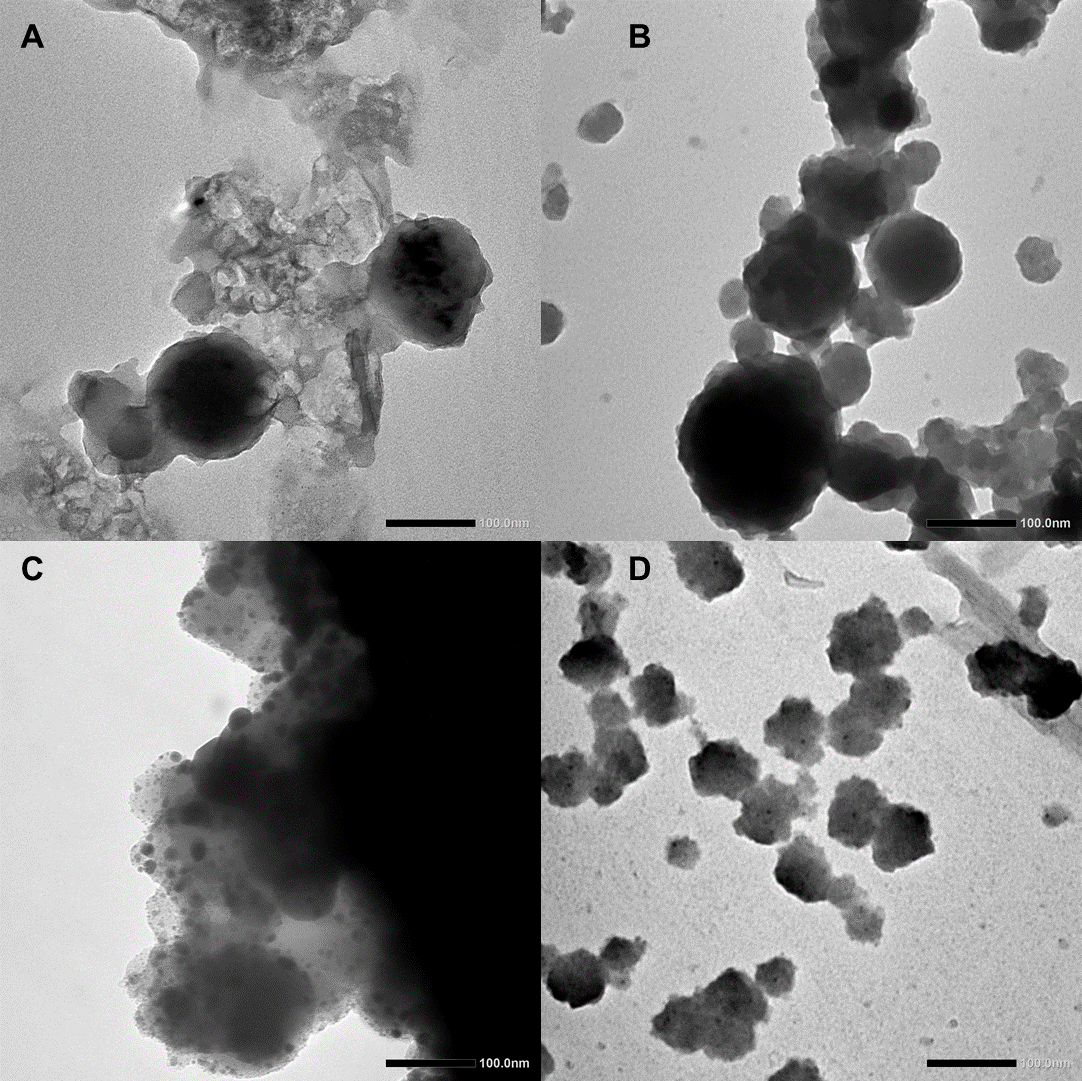


**Figure S11. Binding behaviour of MNP3 (A)** mCherry + MNP (20 mM imidazole) **(B)** mCherry + MNP + 1 mol/l Imidazole (elution, after 2-times washing) **(C)** BSA + MNP (20 mM imidazole) **(D)** BSA + MNP + 1 mol/l Imidazole (elution, after 2-times washing); In all cases the MNP pellet was used and the supernatant discarded. **(C)** and **(D)** should theoretically not contain BSA and **(D)** especially not because of the washing steps; Scale bar is 100 nm.


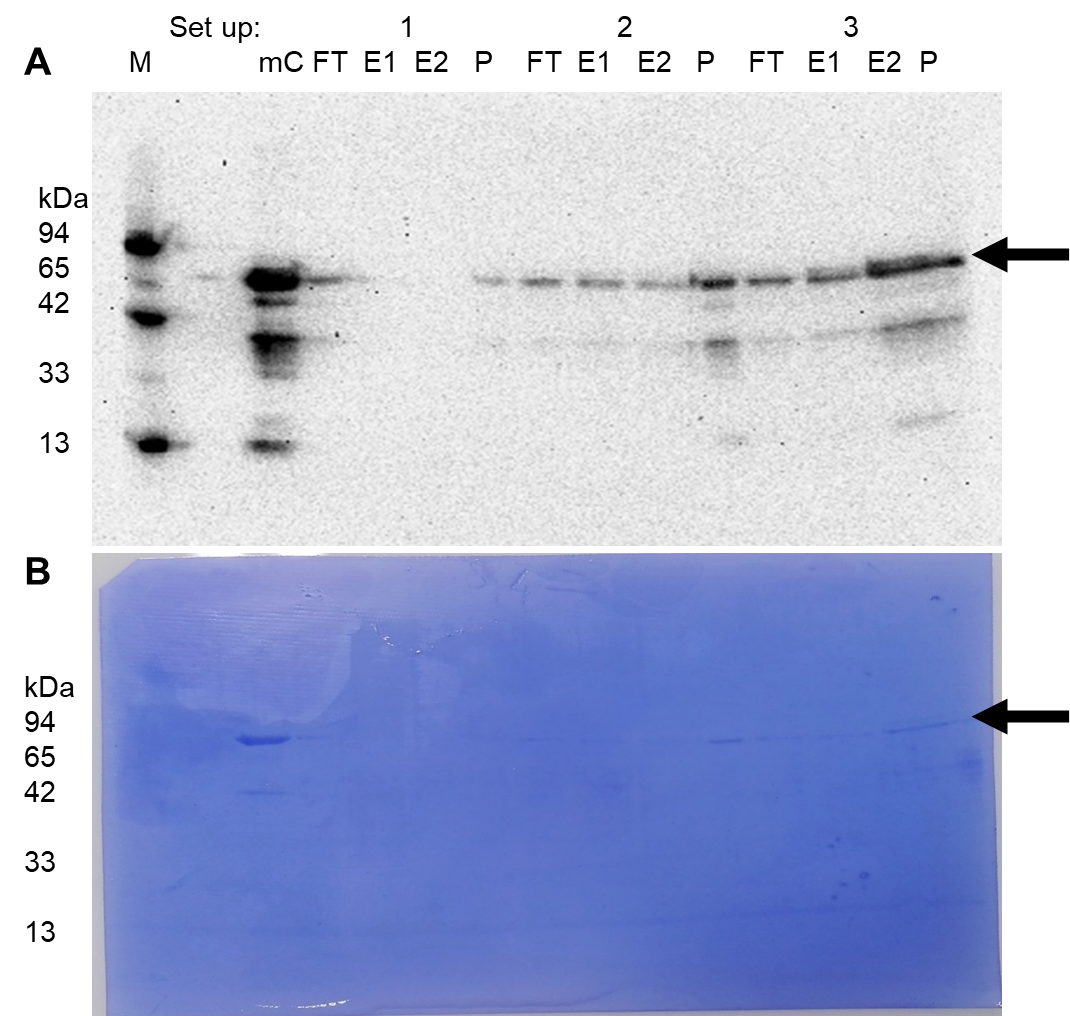


**Figure S12. Binding study of MNP3. (A)** Western blot and **(B)** Coomassie blue stained SDS-PAGE of small binding study with self-made MNP3. Wash buffer contained 20 mmol/l imidazole. The elution for was done with 1 mol/l imidazole, each 50 µl. It was 8 µl sample to 22 µl dilution and on each line 15 µl was loaded. Different set ups were used by the same amount of MNPs (300 µl; 2 mg/ml): 1) 100 µl sample + 550 µl buffer = 12 µg mCherry (protein); 2) 250 µl sample + 400 µl buffer = 30 µg mCherry; 3) 400 µl sample + 250 µl buffer = 48 µg mCherry; FT: Flow through; W1: 1st Wash fraction; W2: 2nd Wash fraction; E1: 1st Elution fraction; E2: 2nd Elution fraction; P: MNPs; M is Marker; Black arrow indicates target protein.

1. Corresponding author: Telephone & fax: +81-54-238-4887.

   *E-mail address:* park.enoch@shizuoka.ac.jp (Enoch Y. Park).

   ^†^ Equal contribution.

   ^#^ Present address: *Institute of Biology and Information Science, Biomedical Synthetic Biology Research Center, School of Life Sciences, East China Normal University, Shanghai 200062, PR China* [↑](#footnote-ref-1)
